# Three-Dimensional Arrangement of Human Bone Marrow Microvessels Revealed by Immunohistology in Undecalcified Sections

**DOI:** 10.1371/journal.pone.0168173

**Published:** 2016-12-20

**Authors:** Birte S. Steiniger, Vitus Stachniss, Verena Wilhelmi, Anja Seiler, Katrin Lampp, Andreas Neff, Michael Guthe, Oleg Lobachev

**Affiliations:** 1 Department of Immunobiology, Institute of Anatomy and Cell Biology, Universität Marburg, Marburg, Germany; 2 Dental Clinics, Histology Laboratory, Universität Marburg, Marburg, Germany; 3 Department of Oral and Maxillofacial Surgery, Marburg University Hospital, Marburg, Germany; 4 Visual Computing, Institute of Computer Science, Universität Bayreuth, Bayreuth, Germany; Medical University Innsbruck, AUSTRIA

## Abstract

The arrangement of microvessels in human bone marrow is so far unknown. We combined monoclonal antibodies against CD34 and against CD141 to visualise all microvessel endothelia in 21 serial sections of about 1 cm^2^ size derived from a human iliac crest. The specimen was not decalcified and embedded in Technovit^®^ 9100. In different regions of interest, the microvasculature was reconstructed in three dimensions using automatic methods. The three-dimensional models were subject to a rigid semiautomatic and manual quality control. In iliac crest bone marrow, the adipose tissue harbours irregularly distributed haematopoietic areas. These are fed by networks of large sinuses, which are loosely connected to networks of small capillaries prevailing in areas of pure adipose tissue. Our findings are compatible with the hypothesis that capillaries and sinuses in human iliac crest bone marrow are partially arranged in parallel.

## Introduction

The microanatomy of the bone marrow has met with increasing interest in recent years. Haematopoietic stem and progenitor cells (HSPCs, [1–3]), plasma cells [4] and T memory lymphocytes [5, 6] have come into focus, because all these cells have been found to reside in special bone marrow microenvironments, so-termed niches. These niches are regarded to be primarily formed by sessile cells of local mesenchymal origin providing essential mediators for survival of the respective cell type. Motile cells such as macrophages may also contribute. The microvasculature of the bone marrow is relatively dense and the relation of niches to this vasculature is regarded to be important. Special niches have been described for HSPCs and for more mature red and white blood cells, located near the endosteum and near the blood sinuses, respectively [7]. However, it is not entirely clear, whether there are distinct micro-compartments for different stages of blood cell development [3, 8, 9]. Especially the location of haematopoietic stem cells in mouse femora is disputed [7, 10]. Niches are, however, also relevant for mature cells, which immigrate into the bone marrow and stay there, such as plasma cells. Exit from the bone marrow also plays a role not only in the course of haematopoiesis [11], but also during recirculation of mature lymphocytes, which at least partially travel through the bone marrow [5, 6, 12, 13]. Bone marrow sinuses form lymphocyte entry and egress portals of decisive function in the physiological setting [12]. This is also important after bone marrow or haematopoietic stem cell transplantation and in spreading of multiple myeloma.

Astonishingly few histological facts are known about rodent and even fewer about human bone marrow microvasculature. Most investigations on bone marrow vessels were done in the femoral growth plates [12, 1] or the femoral diaphysis [7] and the tibia [14] of mice. Flat bones were rarely investigated in this species [15, 16] and may exhibit a unique vasculature. Even in mice the nomenclature applied for different-sized bone marrow microvessels is not consistent. Thus, bone marrow arterioles were described to split up into sinuses [12] or arteries were described to give off capillaries connected to sinuses [3]. Again, other researchers mentioned "sinusoidal capillaries" [17] or arteries, arterioles, capillaries and sinuses [14]. In addition, arteries and two sequential types of microvessel endothelial cells, which most likely correspond to capillary and sinus endothelia, were visualised in mice [1]. Interestingly, it was shown in mice that endothelia located at the arterial side of the microvasculature were associated with less activated haematopoietic stem and progenitor cells (HSPCs] in comparison to sinus endothelia [12, 18]. Arterioles were described to be primarily situated near the endosteum in mouse femora [18]. Several publications found that arteriolar and sinusoidal endothelial cells in mouse femoral bone marrow differ by phenotype [2, 6, 19]. Thus, arteriolar endothelia were described to express Sca-1, but not VEGFR 3 or CD201, while sinus endothelia were positive for VEGFR3 and CD201, but not for Sca-1. Tie-2 (CD202b) was preferentially detected in arterial, but not in sinusoidal endothelial cells [14]. Phenotypic differences and localisation of bone marrow microvascular endothelial cells may not only influence haematopoietic precursors but also mature immunocompetent lymphocytes and a large number of plasma cells. In spite of this fact, a correct histological classification of the single components in bone marrow microvascular networks is lacking in most mouse studies.

It is very likely that bone marrow microvessels in human flat bones, such as the iliac crest, are arranged differently from the microvessels of mouse femoral growth plates. We suppose that especially the amount and distribution of adipose tissue and haematopoietic areas differ according to species, anatomical location and age. In humans, bone marrow microvessels have up to now been primarily demonstrated in small paraffin-embedded biopsies using monoclonal antibodies (mAbs) to CD34 [16, 20–23]. We have now used undecalcified serial sections of a representative iliac crest specimen spanning about 1 cm^2^ and a combination of antibodies against CD34 and CD141 to analyse the 3D arrangement of microvessel endothelium in human bone marrow. Our findings indicate that small-sized and large-sized microvessels, which most probably correspond to capillaries and sinuses, occupy different locations. Both vessel types are at least partially arranged in parallel.

## Results

### Histology

**Fig 1:**
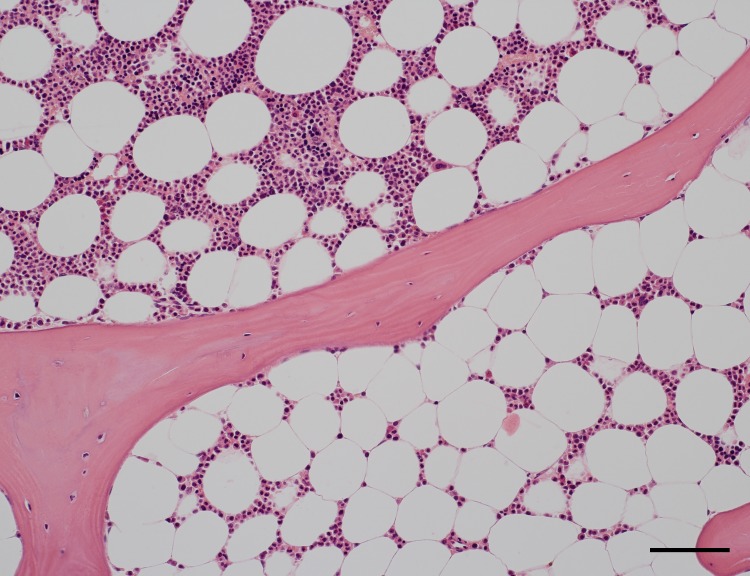
Iliac crest bone marrow stained with hemalum-eosin. Undecalcified section embedded in Technovit^®^ 9100. The quality of the section is equivalent to a decalcified paraffin section. Note the variable density of haematopoiesis within directly adjacent adipose tissue areas. Scale bar = 100 μm.

We show that high-quality sections of undecalcified bone specimens measuring up to 1 cm^2^ can be cut with a modern motorised rotary microtome, if a suitable plastic embedding technique is used (Fig 1). We chose to work with Technovit^®^ 9100, but needed to substantially modify the manufacturer´s protocol for handling this methacrylate formulation. The modifications consist of reducing the viscosity of the methacrylate solution used for infiltration and of prohibiting access of oxygen during polymerisation. In addition, a type C tungsten carbide blade is essential for cutting large numbers of non-compressed bone sections. Only aqueous solutions, but not alcohol, should be used as cutting fluids. Unless high-temperature antigen retrieval is mandatory, Technovit sections can be applied to aminosilane-coated glass slides for de-plasticising and staining.

### Immunohistological detection of bone marrow microvessels

**Fig 2:**
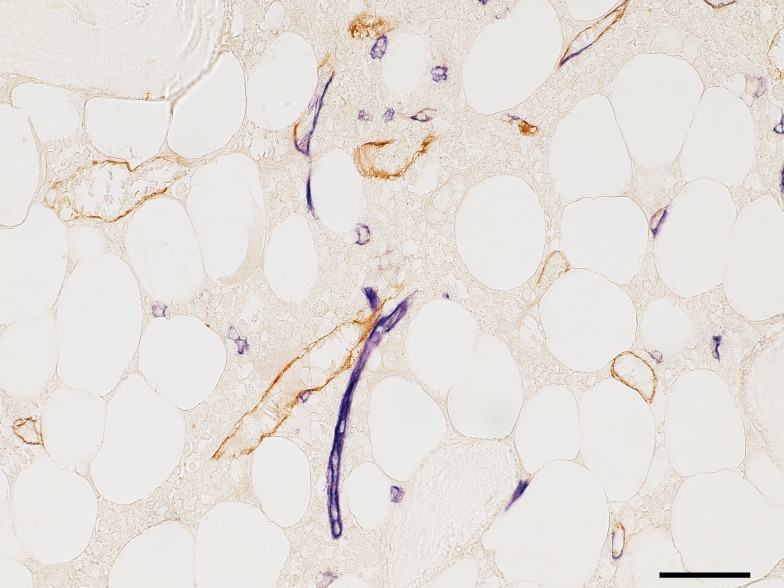
Bone marrow subtractively stained for CD34 (blue) and CD 141 (brown). CD34 is visualised in small microvesssels (capillaries) and is less intensely expressed in large microvessels (sinuses) which exhibit patchy blue and brown staining. Scale bar = 50 μm.

Applying a widely used mAb against CD34 to undecalcified human iliac crest sections from a 53-year-old healthy male patient revealed that CD34 was present in endothelia of small microvessels in the adipose tissue, but was only weakly expressed in large microvessels in haematopoietic areas. We thus additionally tested a mAb against CD141 (thrombomodulin). This mAb stained endothelia in large microvessels, while small microvessels reacted more weakly. Double staining showed that there was a patchy distribution of CD34 or CD141 in large microvessel endothelium (Fig 2). We thus decided to use a mixture of both antibodies for detecting all microvessels. Further analysis of vessels was performed by staining for smooth muscle alpha-actin to detect arterioles.

CD34 was not only expressed in endothelia, but also in round extravascular cells which were interpreted as HSPCs. CD141 also occurred in cells outside microvessels, which might correspond to dendritic cells [24].

### Three-dimensional models of microvessel networks

**Fig 3:**
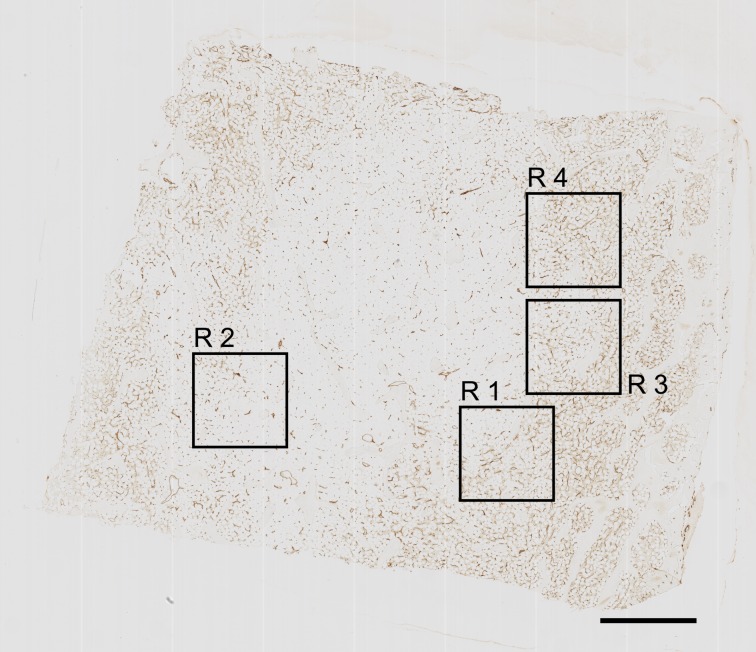
The first of 21 serial sections immunostained for CD34 plus CD141 with regions of interest R1 to R4. Scale bar = 1 mm.

Twenty-one serial sections of the specimen were cut and stained with a mixture of anti-CD34 and anti-CD141. Each stained section was scanned with a Leica SCN 400 automated slide scanning microscope. The bone specimen chosen contained evenly distributed fat cells with intercalated areas of haematopoiesis increasing in size towards the periphery of the specimen. In the centre of the sections almost pure adipose tissue prevailed (Fig 3). Bone trabeculae were also more densely arranged at the periphery, especially at one edge of the specimen which seemed to be connected to a cortical bone region. The specimen chosen was compared to five additional iliac crest specimens and found to be representative with respect to the fact that the haematopoietic tissue was focally distributed in the omnipresent adipose tissue. Our study is based on regions with an intermediate density of haematopoietic cells. Four regions of interest (ROIs), R1-R4, (Fig 3; DOI dx.doi.org/10.5281/zenodo.128996) were chosen. R1 and R2 contained adipose tissue with and without haematopoietic tissue, while R3 and R4 contained only adipose tissue with haematopoiesis. Arteries and typical arterioles were absent in these ROIs as judged from inspection of the immunostained sections.

**Fig 4:**
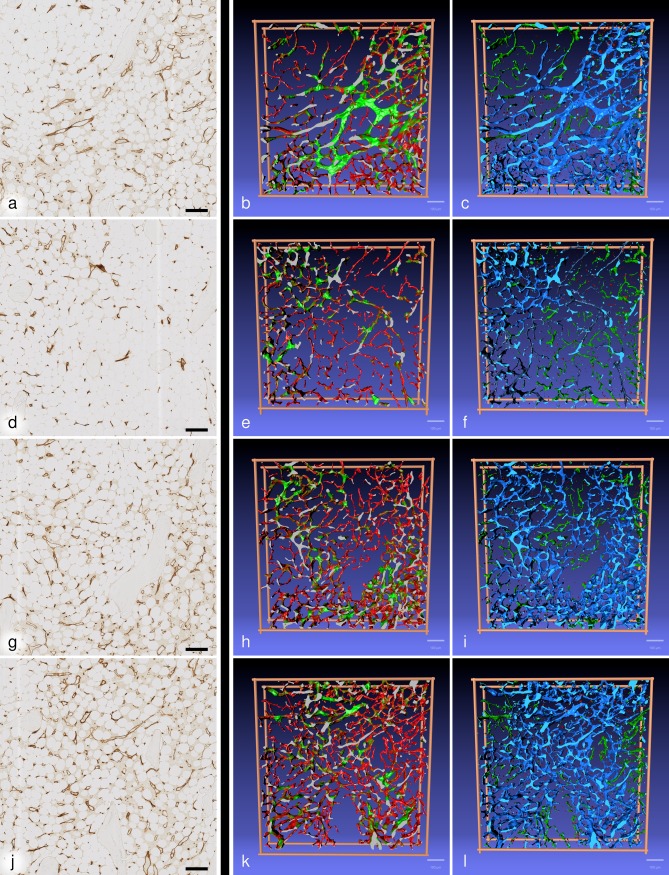
**First scan of R1 (a), R2 (d), R3 (g) and R4 (j) immunostaining in combination with front views of the 3D models coloured for shape diameter (b,e,h,k) and connectivity visualisation (c,f,i,l).** In (b), (e), (h) and (k) a shape diameter below 12 μm is depicted in red and above 30 μm in green. A transition from red to green occurs in microvessels of shape diameters from 12 μm to 30 μm. In (c), (i) and (l) the largest continuous microvessel network is depicted in light blue and all smaller networks are green. In (f) there are several large networks coloured light and dark blue. Small structures sized between 7 μm and 28 μm are red in (c), (f), (i) and (l) and all structures with a diameter less than 7μm in any dimension are omitted. (b,c) R1; (e,f) R2; (h,i) R3; (k,l) R4. Scale bar for all images: 100 μm.

We applied a new multi-resolution non-rigid registration [25], implicating a hierarchy of feature sizes (S1–S4 Videos; DOI: 10.5281/zenodo.129038). Subsequently, volume filtering, 3D reconstruction, mesh filtering, and rendering were carried out. The results were visualised in video format using two types of 3D models (Fig 4).

In the first type of models (Fig 4B, 4E, 4H and 4K; S5–S8 Videos; DOI:10.5281/zenodo.127180), vessels with a shape diameter smaller than 12 μm were depicted in red, vessels with more than 30 μm shape diameter were coloured green and transitional vessels had a brown colour. All non-connected components with a maximum diameter less than 70 μm were excluded. This corresponds to less than 18% of the model surface.

These models demonstrated that networks of small and large microvessels predominantly occupied different locations in the ROIs. The innermost area of R4 (Fig 4k; S8 Video) did, however, form an exception. Single small vessels were sometimes observed inside networks of large vessels, but these small vessels were very short and did not form networks. Connections of small vessel networks to large vessel networks were present, but relatively rare. In such cases, the connecting small vessel often anastomosed to a large vessel network end-to-side with an abrupt change of calibre. Comparison to the immunostaining (Fig 4A, 4D, 4G and 4J) revealed, that networks of small microvessels were primarily associated with areas of pure adipose tissue, while networks of large microvessels were associated with adipose tissue harbouring haematopoietic areas. We thus interpret small microvessels as capillaries and large microvessels as sinuses. R1 contained microvessels of more than 30 μm diameter in a haematopoietic area, which were, however, accompanied by a non-connected network of small microvessels (Fig 4B; S5 Video). We assume that the large vessels represent collecting sinuses or post-sinusoidal venules and the small vessels correspond to capillaries.

Besides capillaries, pure adipose tissue outside the ROIs additionally contained single very large vessels, presumably arterioles and venules. Visual control of the immunostained sections and staining for smooth muscle alpha-actin in directly adjacent sections showed that only very few arterioles with a continuous layer of smooth muscle cells were present within the four ROIs. Thus, almost all vessels visualised corresponded to capillaries and/or sinuses.

The second type of 3D models (Fig 4C, 4F, 4I and 4L; S9–S12 Videos; DOI: 10.5281/zenodo.129038) was created by showing the largest network of connected microvessels in light blue. All other smaller networks were visualised in green and structures smaller than 28 μm, but larger than 7 μm in any dimension, were attributed a red colour. Structures with a diameter less than 7μm in all dimensions were removed. In three of the four ROIs (Fig 4C, 4I and 4L; S9, S11 and S12 Videos) there was one major network of large vessels (light blue) and several unconnected smaller networks (green). R2 (Fig 4F; S10 Video) was an exception, because there were ten unconnected large microvessel networks of similar size. The largest network was thus visualised in light blue colour and all others in dark blue. The red structures primarily represented fused single cells, which appeared elongated in direction of the *z* axis.

This second type of models showed that small microvessels formed networks with few connections to networks of large microvessels in the 21 serial sections. The centre of R4 (Fig 4k and 4L; S8 and S12 Videos) was an exception, because it contained an arrangement of small vessels connected to a network of larger vessels. This arrangement was also detectable by shape diameter function (Fig 4k; S8 Video).

**Fig 5:**
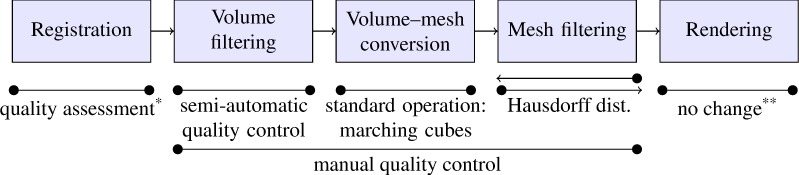
Pipeline of image processing and QC methods, both semi-automatic and manual. * The quality of registration is commented on in Lobachev et al. [25]. ** 3D rendering does not affect the data.

**Fig 6:**
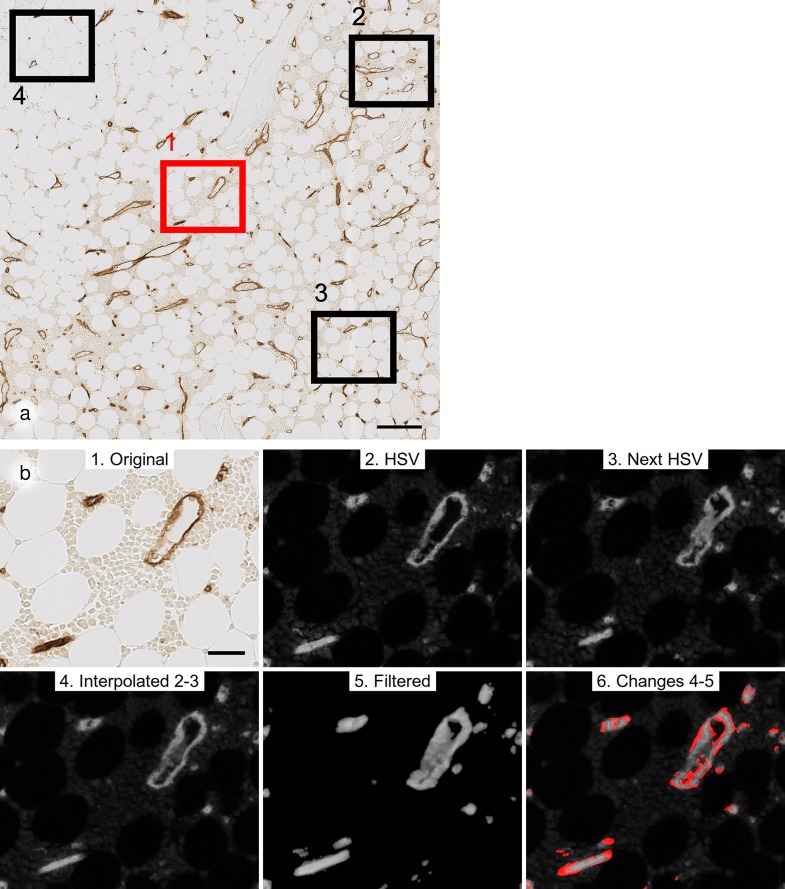
Semi-automatic QC. (a) R 1 containing 4 small regions for semi-automatic QC of volume data processing. The region visualised in (b) is coloured red. Scale bar = 100 μm. (b) Sequence of QC visualised from left to right in upper and lower part of panel: original scan (1), HSV conversion of scan of current serial section (2), HSV conversion of next serial section (3), interpolation between both serial sections: in this case the fourth of seven images is shown (4), final result of all filtering operations of interpolated images (5), areas different between interpolated images and result of filtering coloured red (6). Scale bar = 25 μm.

The 3D models were subject to a thorough quality control (QC, Figs 5, 6A and 6B; S2 Fig, S3 Fig; S14 Video; DOI: 10.5281/zenodo.128861; S15 Video; DOI: 10.5281/zenodo.141383) consisting of two semiautomatic and one manual procedure. The only step that could not be controlled was the initial registration. Quality assessment of this step was described by et Lobachev et al. [25]. All QC methods revealed that blind ends of vessels occurred in the 3D models. Most of these ends were either due to registration errors because of tears in sections or to rather short interruptions of small capillaries after mesh construction (for details of QC see materials and methods, discussion, S1 Text; S2 and S3 Figs; S14 and S15 Videos). Few "real" ends were found when the models were compared to the registered scans. Thus, we are sure that the models presented come very close to reality and loss or addition of major microvessel network components do not occur.

In summary, the microvessels of human iliac crest bone marrow appeared to form a closed vasculature, because erythrocytes were not found outside vessels. All 3D models showed, that the microvessel network at the surface of bone trabeculae did not differ in shape from the network at other locations. Networks of small microvessels, i.e., capillaries, were preferentially associated with pure adipose tissue or adipose tissue with few haematopoietic cells. Networks of large microvessels, i.e., sinuses, were prevailing in areas of more haematopoietically active adipose tissue. Both types of networks had only few connections, i.e., large microvessels did mostly not co-exist together with small microvessels, but they replaced them. Our 3D models provoke a new hypothesis on the arrangement of capillaries and sinuses in human iliac crest bone marrow.

## Discussion

Our study establishes new immunohistological and visualisation procedures for the 3D analysis of human bone marrow morphology in flat bones using large undecalcified sections. The results demonstrate the 3D structure of bone marrow microvasculature in 21 serial sections of the iliac crest and form a basis for further investigations in larger series of sections.

We show that small and large microvessels in human iliac crest bone marrow do not only differ by size but also by preferential expression of CD34 or CD141, although both features do overlap. It is very likely that the small vessels correspond to capillaries and the large vessels represent sinuses. 3D reconstruction of microvessels after combined immunostaining for CD34 and CD141 reveals that both types of microvessels tend to form mutually exclusive networks. This provokes the hypothesis, that capillaries and sinuses are at least partially arranged in parallel. Our findings are thus different from the arrangement published for microvessels of the growth plates or the diaphysis in mouse femora [7, 17, 18]. The video presentations of the 3D models (Fig 4; S5–S12 Videos) indicate that in most areas of the four ROIs smaller microvessels, i.e., capillaries, have only few connections to larger microvessels, i.e., sinuses. Capillaries do, however, accompany larger sinuses or initial venules (Fig 4B and 4C; S5 and S9 Videos). The serial sections investigated comprise areas of pure adipose tissue without haematopoiesis containing only small microvessels and adipose tissue with different amounts of haematopoietic cells associated with networks of large microvessels. Both types of tissue are included in two of the ROIs (Fig 4A–4F; S5, S6, S9, S10 Videos).

We have only stained endothelial cells and thus we cannot unequivocally differentiate very small arterioles (metarterioles) from capillaries in the sequence of the 21 serial sections by morphological inspection. For this purpose double-staining for endothelial and smooth muscle cells would have been necessary. Such a procedure might, however, introduce additional problems into in the models. Thus, staining for smooth muscle cells was performed in the same specimen, but not in the section series.

We define all vessels containing smooth muscle cells in their walls (diagnosed by morphology or immunohistology) as arterioles and hypothesize that these vessels feed capillaries (small microvessels) which only connect to sinuses (large microvessels) near haematopoietic areas. Capillaries and sinuses most probably both drain into initial venules (very large microvessels without smooth muscle cells).

In aged human adult flat and long bones (such as the femoral head), adipose tissue is the prevailing bone marrow component. Haematopoietic areas are focally distributed in the adipose tissue with a tendency to increase towards the compact bone surface. Pure bone marrow adipose tissue only contains arterioles, capillaries (small microvessels) and venules. In directly adjacent focal haematopoietic areas sinuses (large microvessels) and initial venules (extremely large microvessels) prevail, while capillaries are almost absent. Thus, capillaries and sinuses tend to occur in a parallel arrangement in human bone marrow adipose tissue with and without haematopoiesis. Because arterioles are scarce, it is likely, that sinuses are fed by capillaries primarily at the surface of haematopoietic areas, while the major part of the sinus network inside the areas does not coexist with capillaries. We can, however, not directly demonstrate, which vessels feed the sinus network, because 21 sections are not sufficient to follow branching arterioles and because small arterioles were also not detected by immunohistology.

Our hypothesis is derived from a single specimen and thus needs to be regarded with some reservation. We did, however, investigate iliac crest bone marrow specimens from five additional individuals aged 30 to 60 years without performing serial sections or 3D reconstructions. The 2D arrangement of microvessels inside and outside haematopoietic areas was similar in all specimens.

A very special aspect of our method is that we work with undecalcified bone tissue embedded in methacrylate for cutting serial sections of large bone marrow specimens. This has not been reported for Technovit^®^ 9100 before. We avoid the drawbacks of paraffin embedding, such as tedious incubation in EDTA solutions and the uncertainty of analysing the residual calcium content of the specimen. Thus, the overall processing time of the specimens is accelerated, even if the embedding process itself is somewhat more time-consuming than in routine paraffin histology. All steps of our method may be performed with normal laboratory and precision engineering equipment, except polymerisation and sawing. The pressure polymerisation device can, however, be easily replicated (S1 Fig). Modern rotary microtomes are able to produce large high quality sections of undecalcified bone tissue embedded in methacrylate, if a tungsten carbide type C knife and an adequate cutting fluid is used. The distortion is comparable to that of paraffin sections. However, bone tissue is brittle and small cracks in bone trabeculae cannot be entirely avoided. Our embedding method can also be used for sawing and grinding techniques to investigate the integration of metal implants into bone tissue.

Technovit^®^ 9100 is entirely removed from the sections before immunostaining, but this process needs to be carefully monitored. Our method is associated with good preservation of different antigens in the sections. Other investigations have already demonstrated that embedding in Technovit^®^ 9100 is suitable for immunohistology [26–28]. In general, we recommend using a high amplification method for immunostaining, such as a tyramide-enhanced immunoperoxidase procedure. Antibodies need to be individually tested, because preservation of epitopes may be better or worse in comparison to paraffin sections. If a two-component epoxide gluing method is used, high temperature antigen retrieval is also possible in undecalcified bone sections.

The visual analysis of microvessel networks in the models is demanding, although only a rather small number of serial sections is used. We thus try to alleviate the interpretation by introducing two different types of 3D models, one based on vessel shape diameter and the other based on the connectivity of the networks. Both visualisations assist the observer, but the information needs to be carefully interpreted. In Fig 4B, 4E, 4H and 4K and in S5–S8 Videos a vessel shape diameter less than 12 μm in any direction is represented in red colour. Small capillaries are thus nicely highlighted, but broad and extremely flat sinuses also appear red, if viewed from their broad side. There is a rather high number of flat sinuses, extending in direction of the *z* axis. Thus, these models cannot be used to distinguish individual capillaries and sinuses by colour, i.e. by size. They are only intended to help locating the different networks. The second type of models (Fig 4C, 4F, 4I and 4L and S9–S12 Videos) reveals the size of unconnected vessel networks. With exception of R2, the largest network is marked blue and all other unconnected networks of smaller size are green. This visualisation also shows that capillaries are largely separate from sinuses, but it has to be kept in mind that random interruptions of collapsed capillaries and other errors occur. We assume that there need to be several artificial interruptions until networks fall apart, but it cannot be excluded that the real connectivity of the networks in the series is greater than demonstrated. In addition, connections may also be present outside the 21 sections investigated.

Proper thresholding is decisive for the models demonstrated in Fig 4B and 4C. Removing non-connected structures smaller than 70 μm (i.e. smaller than 5% of the main diagonal) is a prerequisite for computing shape diameter function, because the interior of vessels needs to be cleared of precipitates and blood cells. The threshold was determined optically. It also permits better recognition of the overall microvessel network, because it reduces the complexity of the model. Single and merged cells, parts of small non-connected capillaries and parts of vessels isolated by the cut surface are removed, leaving connections between microvessels intact. To demonstrate this fact, we specially controlled the model shown in Fig 4B by selectively visualizing the removed structures in 3D. The difference between the reconstruction mesh and the mesh used in computations of shape diameter function for all ROIs is shown in the repository (DOI: 10.5281/zenodo.163967). The threshold for Fig 4C was 7 μm (i.e. only 0.5% of the main diagonal). It primarily excludes single or fused cells, i.e. structures most probably occurring in only one of the 21 sections.

The image processing pipeline for the 3D models is adjusted to accommodate several problems associated with immunostaining. First, the sectioning procedure may lead to occasional defects in vessel endothelium. Second, staining intensities of endothelia within one and the same vessel may be extremely variable, depending on the angle of cutting. In tangential sections, endothelia leave less immunoreactive material as compared to cross-sections or longitudinal sections and the staining is thus much lighter. These problems are solved by several volume filtering steps.

Background staining does not form a major problem with exception of the background artificially introduced by the scanning microscope. This might have been avoided by fine-tuning the digital processing during data acquisition. Procedures that help with the consistency of the model, such as colour conversion to HSV (hue, saturation, value) and the closing operation (see materials and methods), may connect small separated stained and unstained cells to a close-by blood vessel. The chosen values for closing and further operations are thought to improve the quality of the model while keeping the amount of "fused" cells low. In some sections megakaryocytes have a light brown colour. It is possible to exclude these cells by proper thresholding.

Data processing as performed does not produce smooth vessel surfaces due to cells fused to these surfaces or due to some persisting holes in the walls of larger blood vessels caused by the sectioning or filtering procedures. Further improvements of the processing pipeline may help. However, the aggressive removal of artefacts partially present in the original data might disturb the model and steer it away from reality, for example by increasing the number of "fused" single small cells (see S1 Text for further discussion).

Immersion fixation of the specimens cannot be avoided. In the bone marrow, this procedure obviously provokes the collapse of capillaries, but not sinuses. Thus, the diameter of such vessels may be near 2 μm in adipose tissue. Overall, the diameter differences of blood vessels in the sections are huge. Our volume filtering approach leads to a slightly increased diameter of all blood vessels. This is not conspicuous in large blood vessels, but in capillaries. Nevertheless, the calibre differences between capillaries and sinuses are still clearly visible. In addition, the increase in capillary diameter due to image processing is not really relevant, because it counterbalances the effect of immersion fixation.

Sinuses and fused single cells tend to be elongated in the direction of the *z* axis (S9–S12 Videos). This phenomenon is most likely explained by volume anisotropy. Interpolation and filtering used for anisotropy compensation introduce a directional bias.

In detail, the semi-automatic and manual QC methods revealed that blind ends or interruptions of microvessels were due to one of the following problems listed in the order of the image processing pipeline. 1.) Errors of registration occurred because of disruption or compression artefacts during sectioning of the specimen, especially near bone trabeculae. This error sometimes provoked further errors because of the extreme distortion of adjacent tissue. It was detected by manual QC and occurred infrequently. 2.) Single stained and unstained cells were often fused to the surface of vessels forming artificial buds or short branches. This error was associated with conversion of the original data to HSV and was detected semi-automatically. With respect to unstained cells it was partially due to surface contrast. 3.) Errors primarily associated with filtering of HSV data frequently occurred and were detected semi-automatically. They led to holes or partial loss of the walls of larger vessels. Occasionally this problem provoked interruption of a larger vessel. 4.) Extremely small capillaries were sometimes discontinuous for a short distance and then reappeared. This problem was relatively frequent and occurred during mesh processing. It was shown by manual QC (S15 Video) and by the visualisation of Hausdorff distances (S2 and S3 Figs) 5.) In very few cases did vessels really end inside the series of 21 sections. This phenomenon was detected manually and may represent angiogenesis.

Fig 4C, 4F, 4I and 4L and S9–S12 Videos show, that in addition to blind ends, short elongated red structures were present between the microvessel network, which extended primarily in direction of the *z* axis. These structures were also analysed by manual QC. Most of them were found to be due to volume processing and fusion of single cells located outside vessels. Alternatively, small accumulations of granular extracellular material with a light brown colour also caused short elongated structures. This extracellular material was visible in scans, but not in the original sections. A supplementary discussion of QC results is to be found in S1 Text.

Collapsed capillaries pose the only reconstruction-associated systematic problem that could not be totally remedied. Some of these capillaries are very thin so that short interruptions occur during processing of the surface mesh in spite of high staining intensity. This problem is responsible for most of the interrupted vessels in the final model. The overall structure of the microvessel networks is, however, not altered by this drawback.

Our quality control methods permit direct monitoring of the entire processing pipeline by semi-automatic procedures. In addition, we can manually control for suspicious structures in the final model (Fig 5; S15 Video). In order to achieve models of high reliability, we need to use a large number of data processing procedures. Manual quality control ensures that the reconstruction process does not lead to major artefacts, i.e. selective loss of connections between microvessels of different calibre or accumulation of errors. The microvessels shown in the model are highly congruent to the stained vessels if superimposed on the registered original section (S15 Video). We are not aware of any other models of microvessel arrangement that have been controlled with such stringency.

Others have previously tried to reconstruct microvessel networks from serial immunostained sections of human soft tissues stained for CD34 [29–31]. 3D reconstructions of microvessel endothelia in large normal human bone marrow specimens have not been published before. Previous reports are limited to small human bone marrow biopsies stained for CD34 [16, 23]. We are also not aware of any other 3D reconstruction methods for serial sections that use feature-based non-rigid registration, volume and mesh filtering as well as mesh-based analysis in combination with a comprehensive QC.

Overall, the models indicate that human iliac crests do not only harbour "sinusoidal capillaries" or sinuses, but that capillaries as well as sinuses exist, but preferentially occupy different locations. In addition, staining for endothelia and smooth muscle alpha-actin showed that the distribution of arterioles in the human iliac crest differs from that in mouse femora [18]. In the 21 serial sections we investigated, arterioles were rare. They were not associated with the endosteum and tended to occur in groups, preferentially in areas of adipose tissue without haematopoiesis.

We aim at reconstructing entire sections instead of ROIs and at investigating larger series of sections to find the connections between smooth muscle alpha-actin-positive arterioles and microvessels. However, the information to be handled is extremely large. Reconstructing 21 entire sections will amount to 8.7 x 10^10^ voxels and will necessitate special measures to reduce a *z*-axis bias. We regard this endeavour justified, because human bone marrow does is not only associated with haematopoiesis, but also represents a special type of secondary lymphoid organ and a reservoir for mature granulocytes. Visualising the morphological correlates of these functions in 3D may be achieved by double-staining for microvessels together with lymphocyte populations, granulocytes, macrophages or stromal cells.

## Materials and Methods

### Histological procedures (S13 Video; DOI: 10.5281/zenodo.141383)

#### Bone specimen and fixation

The iliac crest specimen came from a 53-year-old healthy male patient and was left over after retrieval of bone for facial reconstructive surgery. This study was approved by the ethics committee of the Medical Faculty of Marburg University (AZ 35/12). Written informed consent was obtained. The bone pieces were immediately immersed in 3.7% formol in tap water for 24h at 4°C and then transferred via 50% isopropanol to 70% isopropanol for storage. Where possible, larger bone particles were reduced to square tiles of a volume smaller than 1 cm^3^ with less than 3 mm thickness using a diamond band saw. Most particles were, however, irregular in shape. The material was removed from the iliac crest approximately 5 to 10 cm dorsal of the anterior superior iliac spine.

#### Dehydration and defatting

Two tiles each are wrapped in hanging Technovit-resistant mesh tissue in a custom-made support frame designed for small 200 ml screw-cap glass containers. Dehydration is accomplished by immersing 6 to 8 tiles in isopropanol/water 80% once for one hour, then in isopropanol/water 90% twice for one hour and finally in isopropanol 100% three times for one hour. For removal of fat, Xylol is applied once for one hour and subsequently overnight.

#### Infiltration

The bone specimens are embedded in Technovit^®^ 9100 obtained as a kit from Kulzer, Wehrheim, Germany. The infiltration protocol specified in the kit proved inadequate for reproducible embedding results and had to be optimised. The key alterations to the Kulzer procedure are the use of an infiltration solution with reduced viscosity obtained by preparing solution A with only 25 g of polymethylmethacrylate powder instead of 80 g and by introducing an acetone step before infiltration by solution A to optimise penetration into the specimen. Using 80 g of PMMA powder, as recommended by the manufacturer, leads to long stirring times for dissolving the powder and yields a fluid of high viscosity. High viscosity prevents the uniform distribution of Technovit^®^ 9100 in and around hard as well as soft tissues and provokes bubble formation in the centre of the specimen during polymerisation.

**Destabilisation of the base solution:** A 250 ml chromatography column with a glass filter of porosity P4 (pore size 10–16 μm) is filled with 50 g of basic Al_2_O_3_ (Roth, Karlsruhe, Germany, No. X908.1). A maximum of 3 to 4 l of Technovit base solution is slowly passed (at about 300 ml/hour) through the Al_2_O_3_ and subsequently stored in brown glass bottles at -20°C. Porosity P4 is chosen to retain Al_2_O_3_ particles, which may damage the tungsten carbide knife of the microtome.

**Solution A:** Technovit^®^ 9100 is prepared in advance as stock solution A containing 3 g dibenzoylperoxide and 25 g polymethylmethacrylate (PMMA) powder per 500 ml destabilized Technovit base solution. In detail, 250 ml base solution is stirred, 25 g PMMA powder is added and the base solution is filled up to 400 ml. When the solution is clear after about 3 hours of stirring, 3g dibenzoylperoxide is added and base solution is filled up to 500 ml.

**Infiltration procedure:** The low viscosity of solution A improves and simplifies preinfiltration and infiltration. Specimens are transferred from xylol to 100% acetone for 6 hours with one change of acetone and then infiltrated with solution A for 40 hours at 4°C.

#### Polymerisation under pressure

The polymerisation moulds provided by the manufacturer proved totally insufficient, because they were not sealed so that air intruded due to the vacuum arising from polymerisation shrinkage, which can reach 15% by volume. Oxygen slows or even prevents polymerisation of Technovit^®^ 9100. We thus constructed a pressurising device (S1 Fig) with six metal-sleeved Teflon tubes of 16.0 mm internal diameter and about 100 mm length as specimen containers. These containers have sealed mobile bottom and top stoppers to prevent vacuum formation inside. The top stopper of each container always adapts to the surface of the shrinking Technovit solution because it is forced downwards by a spring fixed to a screwed-in metal pressure plate. This principle also prevents bubble formation within the specimen.

#### Preparations for embedding

**Solution B:** Stock solution B is prepared according to the manufacturer´s recommended procedure by first adding 4 ml of N,N,3,5-tetramethylaniline (corresponding to 4.3 g) to 30 ml base solution, then adding 2 ml of 1-decanthiol (corresponding to 1.7 g) to the mixture and finally filling base solution up to 50 ml.

Solution A and B can be stored at -20°C.

**Positioning of specimen tiles for embedding:** The specimen tiles are embedded in an upright position to prevent bubbles caused by polymerisation shrinkage of Technovit^®^ 9100. For this purpose, they need to be held in place by a carrier consisting of the same Technovit used for embedding. Technovit is polymerised in the pressurising device as described below and the resulting methacrylate cylinders of 16 mm diameter and 30 mm length are cut into three shorter cylinders of 10 mm length. On one end face of each cylinder a groove of 3 mm width and 2.5 mm depth is milled into the plastic using a twin-fluted end mill. The groove passes through the centre of the end face. The specimen tile is inserted into the groove thus avoiding the need for glue. The cylinder with the tile is then put onto the bottom stopper and inserted into the polymerisation container. This procedure prevents the specimen from becoming dislodged during the filling process.

#### Embedding protocol

Shortly before starting the embedding procedure, stock solutions A and B are removed from the refrigerator. One part of stock solution B is added to nine parts of stock solution A and stirred with a glass rod for exactly 1 min. Care is taken to quickly proceed with the following embedding procedure. After insertion of the specimen and the bottom stopper, the polymerisation solution is poured into the tube up to a mark and the top stopper is inserted. The stopper contains a screw-hole for the escape of air and for observing whether it touches the surface of the solution during lowering. When this position is reached, a screw is inserted into the hole and tightened. After filling and closing all six tubes in this way, a pressure plate with an affixed spring for each tube is screwed in and tightened until a pressure of about 6 bar (6x10^5^ Pa) has built up.

The pressurising device is then placed into an air-tight plastic bag and put into a refrigerator for 4 days at -14°C to prolong the polymerisation process. After 4 days the device is transferred to an incubator at 37°C for one day to guarantee complete polymerisation. The metal sleeves are then removed from the Teflon tubes and the specimens are pushed out of the tubes with a special lever press.

#### Preparation of embedded specimen tiles for sectioning

The specimens are embedded in an upright position and thus need to be cut from the methacrylate cylinders, turned through 90° and re-fixed in a horizontal position for sectioning with a microtome. The upper face of each cylinder is first made transparent by hand-polishing with increasingly fine grinding paper (grit 1200, 2500 and 3000).

The planes of two parallel cuts and one rectangular cut to be performed with a contact point diamond band saw are then marked on the specimen cylinder using a stereo loupe. Two of these cuts are parallel to the axis of the cylinder, one cut running directly along the specimen surface. A further cut, perpendicular to the axis, separates the specimen from the cylinder. The exposed specimen is then rotated through 90° and glued to the remaining cylinder using isocyanate glue (Loctite 4305, Henkel, Düsseldorf, Germany) for 1 min with subsequent polymerisation in a UV-A polymerisation device (Ivoclar, Schaan, Liechtenstein) for 90 s. To assist cutting with a microtome, the methacrylate embedding material surrounding the specimen is chamfered at an angle of 10°.

#### Quality control of embedded specimens

Before cutting, the specimen surface is polished as mentioned above and the quality of the embedding process, i.e. the absence of bubbles and other artefacts in the specimen, is photo-documented using a modified conventional transmitted light microscope. The modification consists of exchanging the revolving nosepiece below the eyepiece beam splitter for an f = 50 mm objective (Rodagon, Schneider, Kreuznach, Germany) and mounting an SLR camera with a 4/3 chip onto the tube. This arrangement permits the observation of the surface and also the interior of the embedded specimen by using either light reflected at an angle of 30° or transmitted light. Using reflected light necessitates a specimen holder, which may be tilted. For transmitted light the depth of focus is about 2 mm with aperture 16. Thus, the interior of the specimen may be visualised by two photographs.

#### Cutting of hard serial sections

Hard specimens containing bone can only be cut in series, if an up-to-date motorised rotary microtome, such as Leica RM2255, is used. Older motorised microtomes do not provide sufficient mechanical precision and stability. In addition, it is essential that the knife has a type C tungsten carbide blade. This blade has a cutting angle of 27° and thus leads to less compression and distortion of the sections than the conventional type D blade with an angle of 42°. Type D knives inevitably lead to sections shortened by one third in the direction of cutting due to compression. Type C knives are, however, more easily worn than type D knives as they are much less resistant to mechanical lesions during cutting.

The technovit cylinder containing the specimen is fixed to the microtome using a holder for square specimens and a custom-made split brass adapter for cylindric specimens to prevent the embedding plastic from damage caused by repeated mounting and cutting. Alternatively, a proprietary segmented holder for round specimens may be used, but this damages the plastic.

Hard plastic sections need to be cut using a fluid of low surface tension to prevent the sections from rolling up during cutting. The solution is distributed on the section surface and on the blade with a brush before each cut. The lower part of the section, which only consists of embedding plastic is held with fine forceps during cutting and the section is then transferred to a drop of cutting fluid on a slide. In our experience an aqueous solution of 1% BSA (bovine serum albumin) proved optimal for cutting and attaching the sections to silanised glass slides. The manufacturer of Technovit^®^ 9100 recommends alcohol solutions for cutting, but we found that—even if used at high dilution—alcohol provokes cracks in Technovit^®^ 9100 and thus should be totally avoided.

The section on the slide is then covered with a polyethylene film of 25 μm thickness provided by the manufacturer. Starting from one end of the slide the film is pressed onto the slide and attached uniformly by squeezing out the fluid underneath the section. The slides carrying the films are finally placed between two sheets of slide-sized blotting paper to prevent them from damage. They are inserted into a frame-shaped slide press and dried at 50°C overnight under the pressure of 2 Nm, developed by a tightening torque.

**Immunohistology. Primary antibodies:** The following primary monoclonal antibodies (mAbs) were used: MAb QBend10 against CD34 (Dianova, Hamburg, Germany, No. DLN-09135), mAb TM1009 against CD141 (DAKO, Hamburg, Germany, No. M 0617), and mAb asm-1 (Progen, Heidelberg, Germany, No. 61001) against smooth muscle alpha-actin.

The antibodies had been pre-tested on sections of human spleen specimens embedded in paraffin and in Technovit^®^ 9100. They can be used without antigen retrieval.

**Staining Procedures:** Methylmethacrylate is removed from the sections by three 20 min treatments with methoxyethylacetate (MEA). The slides are then transferred to MEA:water solutions of 80%, 50% and 20% for 7 min each followed by two additional 10 min rinses in distilled water.

Single staining, peroxidase technique: Endogenous peroxidatic activity is removed by treating the sections with 0.4 U/ml glucose oxidase/10 mM beta-D-glucose/1 mM NaN_3_ in PBS for 60 min at 37°C. After washing in PBS, MAb QBend10 and mAb TM1009 are mixed in PBS/1% BSA/0.1% NaN_3_ containing 0.003 mg/ml avidin at a final dilution of 1:3000 and 1:60, respectively. Both reagents are applied overnight at 4°C. Then the sections are incubated with the biotinylated secondary antibody of the Vectastain^®^ Elite Kit for Peroxidase (Vector Laboratories, Burlingame, USA, No. BA-9200, via Alexis, Grünberg, Germany) for 30 min at room temperature as recommended by the supplier with a final concentration of 0.02 mg/ml biotin in the solution. Subsequently, the avidin-biotinylated peroxidase complex is prepared according to the instructions and also applied for 30 min at RT. MAb TM1009 needs amplification by tyramide enhancement, a procedure, which leads to increased deposition of biotin by peroxidase-catalysed binding of biotinylated tyramide to antigen-containing areas. For this purpose, biotinylated tyramide is prepared by incubating 50 mg NHS-LC-biotin (Pierce No. 21335) in 20 ml 0.025 M borate buffer pH 8.5 with 15 mg tyramine-HCl (Sigma No. T 2879) on a stirrer overnight. After filtration, this solution is either stored frozen for further use or diluted 1:500 in TBS pH 7.6 containing 0.09 mM H_2_O_2_ and immediately applied to the sections for 10 min at RT. Finally, after washing, the avidin-biotinylated peroxidase complex of the Vectastain kit is applied again as described above and peroxidase reactivity is revealed in brown colour by a diaminobenzidine (DAB) reaction in TBS. The sections are then dehydrated and coverslipped in Eukitt^®^ (Sigma-Aldrich, Steinheim, Germany, No. 03989). A negative control using the Vectastain kit without a primary antibody is included in each experiment. Sections of Technovit-embedded human spleen tissue are used as positive controls. MAb asm-1 against smooth muscle alpha actin was diluted 1:1000. This reagent was used with tyramide amplification and with or without high temperature antigen retrieval.

Single staining, alkaline phosphatase technique: MAbs QBend10 or TM1009 are used at 1:500 or 1:60 dilutions in the same buffer as mentioned above. Instead of the biotinylated secondary antibody and the ABC complex, the ultravision labelled polymer kit (Thermo Fisher Scientific, Schwerte, Germany, No. TL-060-AL) for alkaline phosphatase (AP) or the Vectastain AP elite kit (Vector Laboratories, Burlingame, USA, No. AK-5000, via Alexis, Grünberg, Germany) are applied according to the instructions of the manufacturers. The presence of alkaline phosphatase is detected in blue colour by a standard reaction with naphthol-AS-MX-phosphate in Tris/HCl pH 8.2 and Fast Blue BB salt containing 240 μg/ml levamisole. The slides are coverslipped in Mowiol (polyvinyl alcohol, Sigma-Aldrich, Steinheim, Germany, No. 32.459–0).

Double staining: MAb QBend10 is applied first at 1:500 and visualised with Fast Blue as described above. After washing, the sections are then incubated with mAb TM1009 at 1:60 with detection by tyramide amplification and DAB. Double-stained specimens are coverslipped with Mowiol. Two controls omitting either the first or the second primary antibody are always included in each experiment. This is a subtractive method. Cells stained for the first primary antibody do no longer stain for the second primary antibody.

**Photo-documentation of staining results:** The slides are photographed using a Zeiss Axiophot microscope with a Canon 60D digital SLR camera using the life view function for focussing on a screen.

### Video-documentation of working procedures

S13 Video is produced using a Panasonic Lumix DMC-GH4 camera and a Lumix G Vario 14–140 mm/3.5–5.6 lens as well as a H-ES045 Leica DG Makro Elmarit 45 mm/2.8 lens. Blender 2.7.6 (Blender Foundation, Amsterdam, Netherlands) open source software is used for video editing.

### Imaging Methods

#### Registration and selection of regions of interest (ROIs)

The serial bone sections are scanned using a Leica SCN 400 microscope with a 20x lens which produces an output resolution of 0.28 μm/pixel. The single sections of the series are not aligned. The computer-based processing procedure consists of five consecutive coarse steps: registration, volume filtering and segmentation, volume-to-mesh conversion, mesh filtering including shape diameter function (SDF) processing or visualisation of connected components and rendering (Fig 5).

The complete scanned images of each section are very large, typically 23k x 27k pixels. We assumed the thickness of a single section to be 7 μm, which is the average in a thickness range from 5 to 10 μm. The images need to be registered before any further processing can take place. Registration was done according to the procedure of Ulrich et al. [32], which represents a sparse single-resolution non-rigid registration method. Non-rigid registration was introduced to mend the distortion by the sectioning procedure. We deem this registration a coarse one.

The ROIs can only be selected after coarse registration (Fig 3). The aim was to find interesting regions partially containing adipose tissue as well as haematopoietic areas and lacking artefacts through the whole stack of 21 sections. The size of ROIs selected was 4k x 4k pixels. After coarse registration we applied a refined registration method. This method represents a sparse multi-resolution non-rigid registration [25]. A hierarchy of feature sizes was used to successfully register not only the large features (corresponding to large blood vessels or other large structures such as trabeculae), but also smaller features that correspond to microvessels. After the registration we trimmed the edges of the image stack to obtain cut surfaces on the sides. The final resolution was 3.5k x 3.5k pixels in 21 images (S1–S4 Videos; DOI: 10.5281/zenodo.129038). The registration software was custom-written. It is available as part of the supplemental material published by Lobachev et al. [25].

#### Interpolation and conversion of colour data

At this point we still work with the original image data corrected for distortions. For further processing, including the interpolation, we convert the RGB (red, green, blue) data of the scanned serial sections stained in brown to the saturation channel of the hue saturation value (HSV) colour space. Basically, in the HSV saturation channel, the image is light where the saturation is high in stained cells and dark in non-stained areas. We use single-channel images to reduce data volume. This reduction is required for subsequent compensation of anisotropy by interpolation that increases the number of images from 21 to 140. We basically reduce data size from 4.79 GB to 1.60 GB in uncompressed form by using one colour channel with 3500 x 3500 x 140 voxels. Alternative and potentially simpler transformations are theoretically possible. For example, the inverted red channel of the RGB data alone would also produce a good result.

The resolution of the scan in the *xy* plane (i.e., in the scanning plane) is 0.28 μm/pixel. However, the resolution in the direction of the *z* axis (i.e., the thickness of the section) is only 7 μm/section. This high anisotropy would prevent us from obtaining optimal results. Hence, we interpolate between each pair of consecutive sections. We use dense optical flow [33] to obtain the "movement" between section *i* and section *i+1* for all *i* from 1 to 20. This motion information is then used to generate intermediate images between two sections. The resolution in the direction of the *z* axis thus becomes 1 μm/interpolated slice. The resulting "stack" of 140 images is the input volume data for further processing. This operation is performed with a custom-written software based on the open source library OpenCV (version 3.1.0, [34]).

#### Filtering

To enhance the final result, we apply a set of filters to the volume data. Basically, we use a sequence of simple image processing operations aimed at obtaining better input data for the 3D reconstruction. In this step, we remedy intensity changes still visible in the images due to tangential sectioning of stained vessels or cells or to small interruptions in the contours of blood vessel walls.

The image processing operations consist of the following steps. First, we threshold the volume: all pixels with intensity below 70 (of 255) are set to black. This is a coarse form of segmentation. Next we apply a 3D greyscale closing operator with kernel radius of 10 voxels. Then this closing filter is used to mend at least some missing parts of blood vessel walls. We need to eliminate the inner contours of the vessel walls, to permit correct computation of large vessel diameters in later steps. The hole fill method is one procedure to approach this problem. Further procedures to remove remnants of double contours are executed at the mesh processing stage. After applying a close hole filter we perform a greyscale 3D dilating operation with a radius of 3 voxels. Next, another closing filter with a radius of 5 voxels is applied. Finally, the image is blurred with a Gaussian blur procedure with sigma value 0.33 to smooth the surface of the reconstructed blood vessels.

In summary, six filters, namely Threshold 70, Close 10, Fill hole, Dilate 3, Close 5 and Blur 0.33, are applied to the initial interpolated volume.

All these operations are performed in the open source software 3D Slicer (version 4.5.0–1 r24735, [35]). The changes which the original data undergo are shown for 4 small regions within each of the four large ROIs (Fig 6A and 6B; S14 Video; DOI: 10.5281/zenodo.128861). This quality control procedure permits visual comparison of the original aligned section (Fig 6B1), the HSV conversion (Fig 6B2) and (Fig 6B3), the interpolation (Fig 6B4), and the final result of filtering (Fig 6B5). Differences between Fig 6B4 and Fig 6B5 are highlighted in Fig 6B6.

#### Mesh construction

Next, we apply the marching cubes algorithm [36] to convert the volume representation to a mesh. We start with a volume data set, similar to a usual 2D image. We have voxels, "3D pixels", for each point in the direction of the *x*, *y* and *z* axis. After performing the marching cubes algorithm, we obtain a "mesh", a network of triangles that basically corresponds to surfaces of constant intensity (so-termed iso-value) in the volume. Basically, our desired result is a network of triangles corresponding to the surface of blood vessels we aim to depict. Meshes are the standard data representation form in computer graphics. The marching cubes operation was also performed using 3D Slicer.

The output mesh is large (typically about 1.5 GB) and direct processing is troublesome. Application of smoothing and decimation methods build-in in 3D Slicer did not produce the desired result as they greatly impacted the quality of the output mesh. We thus apply a series of filtering operations to reduce data size and enhance the quality of the final visualisation. First, we remesh and heal the initial mesh using PolyMender (version 1.7.1; [37]), then we apply 10 iterations (the default setting) of the Taubin smoothing filter [38]. The goal of "Taubin smooth" is to reduce the unevenness of the blood vessel surface without disturbing object size. This step happens in MeshLab (version 1.3.3, [39]). Next we proceed to "solidify" and remesh the data again. We aim for a representation of blood vessels as solid forms even though we use the mesh, a surface-based representation. For this purpose, we apply an automatic processing operation available in MeshMixer (version 11.0.544, [40]). We also use this software to improve our presentation of cut surface planes. The final meshes for all video presentations are deposited as DOI: 10.5281/zenodo.129037.

#### 3D-Models

3D models are presented using two different colour codes. In the first type of models (Fig 4B, 4E, 4H and 4K; S5–S8 Videos) we need to totally remove all inner contours inside the mesh network. We map each vertex with its volumetric obscurance factor [41], basically noting down how visible the particular point is. Then we remove points (actually: vertices with connected edges and faces) that have low visibility. In order not to disturb the actual mesh surface by this procedure we choose the selection threshold in a way preventing deletion of vertices on the mesh surface. Hence, we delete only vertices inside the closed structure. Subsequently, we eliminate all non-connected components inside and outside blood vessels the size of which is less than 5% of the space diagonal of the model, i.e. components with a largest diameter less than 70 μm.

The resulting mesh is processed using the so-termed shape diameter function (SDF, [42]). The basic idea of SDF is simple: from each point (vertex) in the mesh, rays are cast in varying directions. When a ray encounters a surface in the mesh, it stops. The shortest of all rays is recorded at the vertex. If we additionally utilise the normals, i.e., if we know the "inside" and "outside" of the mesh, and if rays are only shot inside the mesh, we obtain the diameter information at each vertex, the final result of SDF. We use the implementation of SDF from MeshLab.

To visualise the SDF data, we colour all vertices red if the SDF value is under 12 μm, and green, if it is above 30 μm. Intermediate values are shown as a gradient from red to green. (Fig 4B, 4E, 4H and 4K; S5–S8 Videos). The gradient is centred at 16.5 μm. The corresponding colour scheme for MeshLab is available at DOI: 10.5281/zenodo.127180. In order to establish sectioned surfaces of the entire model, we replace the colour information at the sectioned surface by light grey using a custom-written mesh segmentation script.

The second type of models (Fig 4C, 4F, 4I and 4L; S9–S12 Videos) demonstrates the largest non-connected microvessel network in blue colour, smaller non-connected networks in green and small components sized 7 μm to 28 μm in their largest diameter in red. The smallest components (smaller than 7 μm in largest diameter) are discarded using a filter in MeshLab. The cut surface of the ROI is coloured light blue. The connectivity test is a simple mesh-based analysis technique, which basically detects connected triangles.

S5 Video to S12 Video were produced using commercial Cinema 4D software (version R14.0429, MAXON Computer GmbH, Friedrichsdorf, Germany).

#### Quality control of the three-dimensional models (Fig 5)

In order to control the quality of the 3D models generated from the four ROIs, we established two semi-automatic and one manual quality control (QC) methods. In detail, we devised two semi-automatic QCs for volume filtering and mesh filtering and a manual QC covering all operations from the result of registration to the input into the video rendering program.

For semi-automatic QC of all volume-related procedures after the registration step we designed a sequence of optical visualisations in three to four small regions per ROI. In these small regions we tried to detect errors caused by processing of the volume data, consisting of conversion to the saturation channel of the HSV colour space, interpolation between two consecutive sections in HSV and application of several types of filters (see also "filtering", Fig 6A and 6B; S14 Video; DOI: 10.5281/zenodo.128861).

The second semi-automatic QC method consisted of measuring Hausdorff distances between meshes (S1 Text, S2 and S3 Figs). It was also used for repair purposes. For the processing of cut surfaces we need to resort to algorithms that may significantly alter further parts of the mesh surface. Among other operations, we remesh and solidify the mesh representation of the vasculature to produce filled, flat and segmented cut surfaces. A control step is mandatory afterwards to ensure consistency. We automatically measure Hausdorff distances to detect parts of the marching cubes output mesh that are lost during the processing and then add the lost parts to the processed mesh.

The Hausdorff distance computation is a unidirectional representation of distances from one mesh to another [43]. At each vertex of the first mesh rays are shot in all directions. As soon as a ray intersects the surface of the second mesh, the ray is terminated. The length of the shortest ray is noted at the vertex. This procedure defines the distance between two meshes. However, if a component is not present in the first mesh, a distance cannot be determined at that point. For this reason, we need to compute Hausdorff distances in both directions. A large distance from the first mesh to the second identifies structures present in the first mesh and lacking or extremely different in the second mesh and vice versa.

In detail, we compute Hausdorff distances between the result of PolyMender on the marching cubes mesh (first mesh) and the result of the last step of our pipeline (second mesh) and vice versa. All structures from the first mesh that are more than 10 μm away from the second mesh are added to the second mesh. After this procedure short discontinuities may still be present in the repaired blood vessels, but larger parts of microvessels are no longer missing, unless registration or volume processing errors have occurred. The computation of Hausdorff distances provides a reliable tool to control the second part of the reconstruction procedure as demonstrated. We performed manual QC (S15 Video, see below) of blind vessel ends in the four models visualising Hausdorff distances in forward direction (S2 Fig) and found that any missing structures were either correctly detected by the method or were due to the first and not to the second part of the processing pipeline.

With respect to manual QC, we hypothesised that bone marrow microvasculature represents a closed system and sampled 10 vessels per ROI, which had blind ends in the 3-D-models and thus did not reach a second surface. In addition, we controlled single small elongated structures located between the microvessels (shown in red in Fig 4C, 4F, 4I and 4L and S9–S12 Videos). In order to obtain more information about such unexpected structures, we devised a special program which permitted capturing selected structures in the mesh representation of each ROI and projecting these structures either onto single registered scans or onto the entire series of scans. A three-dimensional impression emerges when the view vector is tilted by the user (S15 Video; DOI: 10.5281/zenodo.141383).

## Supporting Information

S1 Fig Pressurising device for polymerisation of Technovit^®^ 9100. Reprinted from [28] under a CC BY license, with permission from S. Karger AG, Basel, Switzerland, original copyright 2013.
(DOC)

S2 Fig **Hausdorff distances from first to second mesh in R1 (a) to R4 (d).** Blue represents identity of both meshes, red indicates a distance of 10 μm or more.
(TIFF)

S3 Fig **Hausdorff distances from second to first mesh in R1 (a) to R4 (d).** Blue represents identity of both meshes, red indicates a distance of 10 μm or more.
(TIFF)

S1 Table Hausdorff distances from first mesh to second mesh (with cut surfaces) (XLS)

S2 Table Hausdorff distances from second mesh (with cut surfaces) to first mesh. (XLS)

S1 Text Discussion of QC results. (DOC)

S2 Text Repository listing. (DOC)

S1 Video Overlay of all 21 immunostained sections of R 1. (MP4)

S2 Video Overlay of all 21 immunostaiend sections of R 2. (MP4)

S3 Video Overlay of all 21 immunostained sections of R 3. (MP4)

S4 Video Overlay of all 21 immunostained sections of R 4. (MP4)

S5 Video 3D model of R1 stained for shape diameter function. Shape diameter below 12 μm is depicted in red and above 30 μm in green.
(MOV)

S6 Video 3D model of R2 stained for shape diameter function. Shape diameter below 12 μm is depicted in red and above 30 μm in green.
(MOV)

S7 Video 3D model of R3 stained for shape diameter function. Shape diameter below 12 μm is depicted in red and above 30 μm in green.
(MOV)

S8 Video 3D model of R4 stained for shape diameter function. Shape diameter below 12 μm is depicted in red and above 30 μm in green.
(MOV)

S9 Video 3D model of vascular network connectivity in R1. The largest continuous network is depicted in light blue and all smaller networks are green. Small structures between 7 μm and 28 μm are coloured red.
(MOV)

S10 Video 3D model of vascular network connectivity in R2. The largest continuous network is depicted in light blue, the second to tenth largest networks are in dark blue, and all smaller networks are green. Small structures between 7 μm and 28 μm are coloured red.
(MOV)

S11 Video 3D model of vascular network connectivity in R3. The largest continuous network is depicted in light blue and all smaller networks are green. Small structures between 7 μm and 28 μm are coloured red.
(MOV)

S12 Video 3D model of vascular network connectivity in R4. The largest continuous network is depicted in light blue and all smaller networks are green. Small structures between 7 μm and 28 μm are coloured red.
(MOV)

S13 Video Methods of specimen processing and staining for serial Technovit^®^ 9100 sections. (MP4)

S14 Video Sequence of QC in the small region depicted in Fig 6A and 6B. The remaining 13 videos are found in the repository (DOI: 10.5281/zenodo.128861). For each small region, the following processing steps are visualised from left to right in the upper and lower part of the panel: original registered data (1), HSV data conversion (2), HSV data conversion of next serial section (3), interpolations between both serial sections resulting in 7 images (4), final result of all volume filtering operations of interpolated images (5), areas differing between interpolated images and the result of filtering visualised in red colour (6).
(MOV)

S15 Video Demonstration of the custom-designed program for manual QC. (MP4)
