# Supplementary material for: Three-Dimensional Arrangement of Human Bone Marrow Microvessels Revealed by Immunohistology in Undecalcified Sections
Source: PLoS One. 2016 Dec 20;11(12):e0168173. doi: 10.1371/journal.pone.0168173 (PMC5172587; doi:10.1371/journal.pone.0168173)
Supplement: S1 Fig — Reprinted from [28] under a CC BY license, with permission from S. Karger AG, Basel, Switzerland, original copyright 2013. (DOC) [file pone.0168173.s001.doc]

**
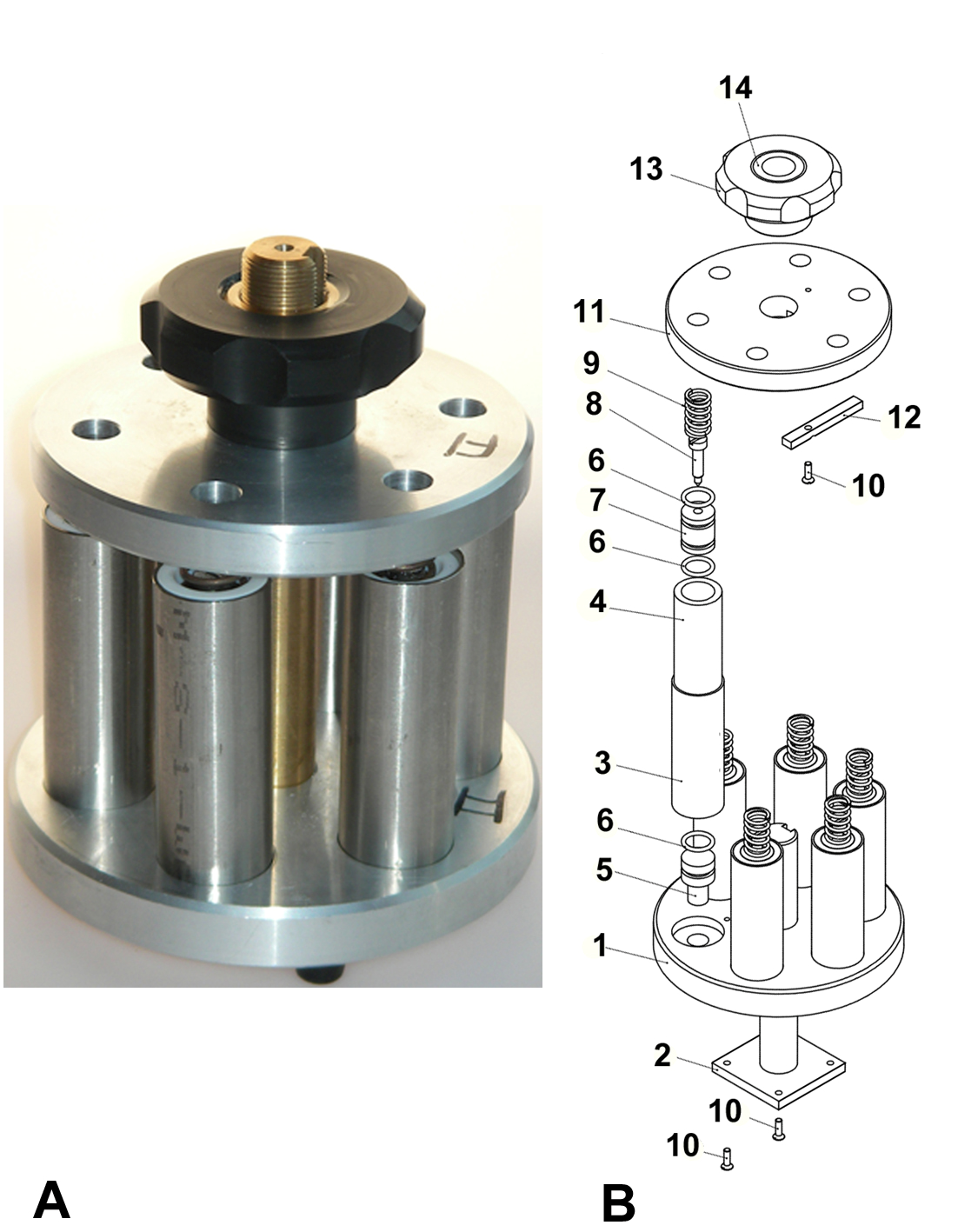
**

**Online Suppl. Fig. 1.** External aspect **(A)** and technical drawing **(B)** of the newly designed device for polyme­rizing Technovit® 9100. Components in **(B)**: **1** - bottom pressure plate, **2** - drawbar, **3** - steel jacket, **4** - pressure cylinder, **5** - piston, **6** - O ring, ISO 3601, 12mm x 2mm, **7** - vented piston, **8** - venting screw, **9** - compression spring, ISO 10243, 1.6mm x 12.5mm x 21.5mm, **10** - M3 countersunk slot head screw, ISO 2009, 10mm, **11** - upper pressure plate, **12** - pilot pin, **13** - screw for upper pressure plate, **14** - thread insert

Items **4**, **5**, and **7** consist of teflon.
