# Supplementary figures and images for: Three-Dimensional Arrangement of Human Bone Marrow Microvessels Revealed by Immunohistology in Undecalcified Sections
Source: PLoS One. 2016 Dec 20;11(12):e0168173. doi: 10.1371/journal.pone.0168173 (PMC5172587; doi:10.1371/journal.pone.0168173)

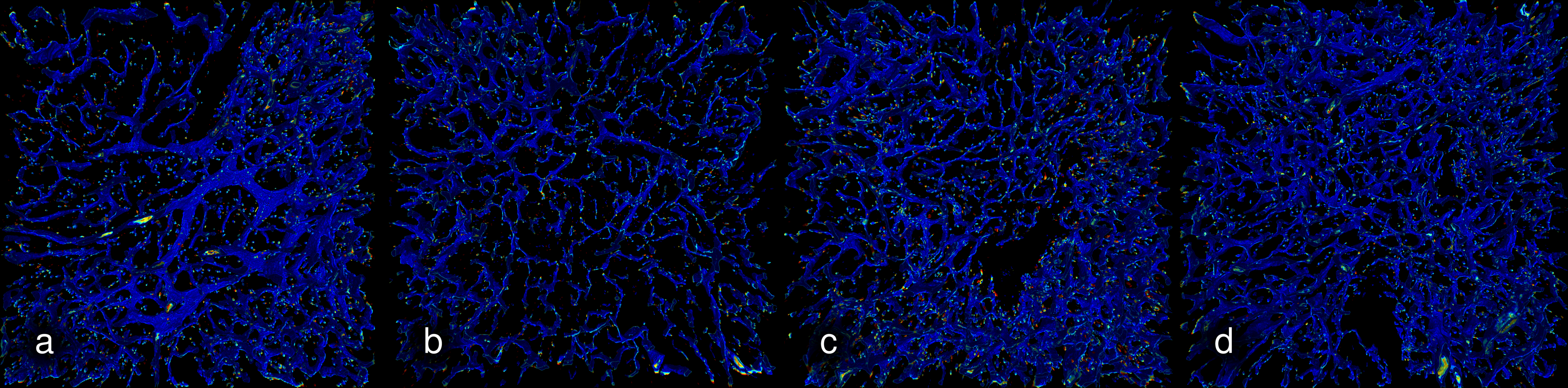

Supplement: S2 Fig — Hausdorff distances from first to second mesh in R1 (a) to R4 (d). Blue represents identity of both meshes, red indicates a distance of 10 μm or more. (TIFF) [file pone.0168173.s002.tiff]

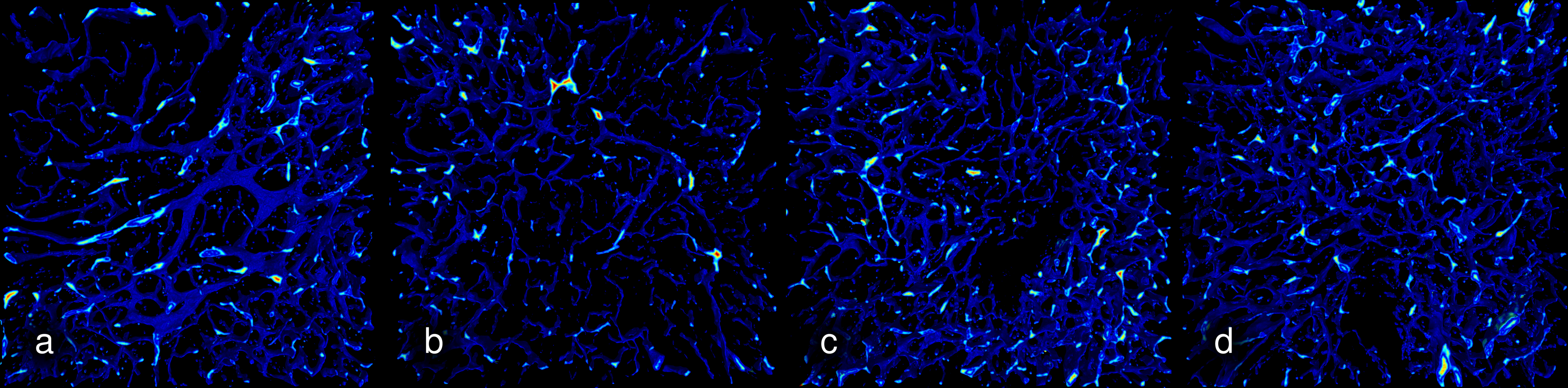

Supplement: S3 Fig — Hausdorff distances from second to first mesh in R1 (a) to R4 (d). Blue represents identity of both meshes, red indicates a distance of 10 μm or more. (TIFF) [file pone.0168173.s003.tiff]
