# Supplementary material for: Three-Dimensional Arrangement of Human Bone Marrow Microvessels Revealed by Immunohistology in Undecalcified Sections
Source: PLoS One. 2016 Dec 20;11(12):e0168173. doi: 10.1371/journal.pone.0168173 (PMC5172587; doi:10.1371/journal.pone.0168173)
Supplement: S1 Text — (DOC) [file pone.0168173.s006.doc]

**S1 Text: Discussion of QC results**

Errors of registration due to sectioning artefacts can neither be avoided nor con­trolled, because the arrangement of the microvessels and trabeculae before section­ing of the specimen is unknown. We do, however, regard the novel registration meth­od applied [25] as optimal, because it uses automatic detection of landmarks of vari­ous scale allowing to register both small and large image elements. In addition, the method functions for features with a high degree of self-similarity, typical of haema­topoietic bone marrow. Lobachev et al. [25] corroborate this by a sequence of quality assessments.

Fusion of cells partially result from the colour space conversion procedure where the saturation does not decrease as fast as the immunohistological staining, i.e., in the narrow spaces between blood vessels and uncoloured cells. The colour intensity of "unstained" cells is sometimes artificially increased by the scanner so that their satura­tion is above the segmentation threshold. A higher segmentation threshold is not possible during volume filtering, as this might produce even more holes and blind ends in the vasculature. Another cause for cell fusion was due to the fact that two points are connected by the closing filter if the distance between them is less than 10 voxels (2.8 x 2.8 x 10 µm).

For quality control of the second part of the processing pipeline, the results of march­ing cubes processing (the "first" mesh) and the final mesh after filtering and repair (the "second" mesh) are compared. S2 and S3 Figs. show the Hausdorff distances between both meshes in forward or reverse direction projected either onto the first (S2 Fig.) or the onto the second mesh (S3 Fig.) as rainbow colours (blue represents zero, red represents 10 µm and more). Cut surface planes are missing from the first mesh and have been artificially introduced into the second mesh.

The Hausdorff distances from the first mesh to the second show that the meshes have a very similar shape in most regions (blue in S2 Fig.). There are, however, com­ponents of the cut surfaces and smaller structures outside vessels in the first mesh, which are absent from the second mesh thus leading to large distances in forward direction. All fragments from the decimated "first" mesh with larger Hausdorff dis­tances have been included in the repair process. S2 Fig. (a–d) shows Hausdorff dis­tances plotted on the first mesh for R1 to R4. The statistic­al evaluation of these dis­tances is given in S1 Table. Average values range from about 1.0 µm to 1.6 µm, the root mean square (RMS) as a measure of variance lies between 1.6 µm and 2.6 µm. If the space diagonal of the fi­nal mesh is taken as a 100% reference, the maximal distance (which occurs only infrequently) amounts to less than 4%. The mean dis­tances are between 0.07% and 0.18% of the space diagonal and the RMS also ranges from 0.11% to 0.19%.

The results of Hausdorff distance computation in reverse direction, i.e., from the second mesh to the first one also show good congruency of both meshes. Typical distances are close to zero (S3 Fig. a–d), the mean distance is in the range of 0.42 µm to 0.65 µm, the maximum is 13.0 µm (S2 Table). In this direction the average Hausdorff distances range from 0.03% to 0.05% of the space diagonal and the max­imal distances are below 0.94%. RMS is also below 0.06%. The distances at cut ves­sel surfaces are larger because of the artificial planes introduced, which increase the average distance. In fact, colours other than blue only appear at these surfaces in S3 Fig. In general, Hausdorff distances are smaller in reverse direction.
