# Supplementary material for: Three-Dimensional Arrangement of Human Bone Marrow Microvessels Revealed by Immunohistology in Undecalcified Sections
Source: PLoS One. 2016 Dec 20;11(12):e0168173. doi: 10.1371/journal.pone.0168173 (PMC5172587; doi:10.1371/journal.pone.0168173)
Supplement: S2 Text — (DOC) [file pone.0168173.s007.doc]

**Repository Zenodo**

Overview videos (S1-S4 and S9-S12) at higher resolution: DOI: 10.5281/zenodo.129038

Registered data sets for R1-R4: DOI: 10.5281/zenodo.128996
Videos S5-S8 at higher resolution: DOI: 10.5281/zenodo.127180

Videos S13 and S15 at higher resolution DOI: 10.5281/zenodo.141383
Final filtered volume data: DOI: 10.5281/zenodo.128884

Final meshes: DOI: 10.5281/zenodo.129037
Semi-automatic QC for 14 small regions: DOI: 10.5281/zenodo.128861

Differences between both types of models for the four ROIs visualised in red colour including colour scheme: DOI: 10.5281/zenodo.163967
